# Italian Validation of the Touch Avoidance Measure and the Touch Avoidance Questionnaire

**DOI:** 10.3389/fpsyg.2020.01673

**Published:** 2020-07-23

**Authors:** Laura Casetta, Luca Rizzi, Marcello Passarelli, Giorgio Arcara, Raffaella Perrella

**Affiliations:** ^1^Associazione Centro di Psicologia e Psicoterapia Funzionale, Istitituto S.I.F., Padua, Italy; ^2^Institute of Educational Technology, National Research Council, Genoa, Italy; ^3^IRCSS San Camillo Hospital, Venice, Italy; ^4^Department of Psychology, University of Campania “Luigi Vanvitelli,” Caserta, Italy

**Keywords:** touch avoidance, social touch, ordinal CFA, non-verbal communication, Italian validation

## Abstract

Social touch is essential in relationships and well-being, but the unique personal experience of touch is not assessed and taken into account in health and social care services. The pleasantness of gentle stroking is influenced by gender, toucher genre, toucher familiarity, culture, and age. Moreover, pleasantness is influenced by touch avoidance, the attitude toward interpersonal touch. The aim of this article is to present the translation, adaptation, and validation in Italian of two scales to measure touch avoidance. For translation and validation, we selected the most used scale, the Touch Avoidance Measure (TAM) and a more recent scale, the Touch Avoidance Questionnaire (TAQ). Confirmatory factor analyses reported good model fit for the TAM [comparative fit index (CFI) = 0.947, Tucker–Lewis index (TLI) = 0.940, root-mean-square error of approximation (RMSEA) = 0.065] and excellent model fit for the TAQ (CFI = 0.954, TLI = 0.950, RMSEA = 0.058). Internal consistency was high for all subscales, except the TAQ “Stranger” subscale. One-month test–retest reliability ranged from 0.67 to 0.90 for each subscale. Lastly, convergent validity between the TAM and TAQ was also found to be high. We conclude that the TAM and TAQ can be used to assess touch avoidance with Italian samples. The instrument can be used to support healthcare professionals and to assess attitudes toward touch in individuals with interpersonal difficulties.

## Introduction

Touch is one of the most important senses for survival: it is one of the first to develop in the maternal womb and the most developed at birth (Hertenstein et al., 2006). Touch has several functions: it helps to discriminate the location of a stimulus on the skin surface, to examine objects haptically, and to manipulate them. Touch also serves the function to shape an integrated sense of our body (Serino and Haggard, 2010). Moreover, touch has a communicative function, as distinct emotions can be communicated and correctly decoded through touch (Hertenstein et al., 2006, 2009).

In addition to this, touch in the two last decades has been recognized for its important social function, to the point of calling the skin a “social organ” (Morrison et al., 2010). This highlights the importance of one characteristic of the sense of touch: its pleasantness. Pleasantness of touch has been explained through the *social touch hypothesis* (Olausson et al., 2010) a theory that explains that slow gentle stroking is pleasant because it is important in close affiliative interactions.

The pleasantness of touch is due to the C-tactil (CT) afferents pathway, an unmyelinated, slow conducting (0.6–1.3 m/s), low-threshold mechanoreceptor that only innervates hairy skin (Vallbo et al., 1999; McGlone et al., 2014). In addition to the neurophysiological basis of pleasantness connected to gentle stroking, personal and contextual factors influence the response of pleasantness. For example, receiving a gentle stroke by an undesirable toucher changes the pleasant experience to disgust (Ellingsen et al., 2016). Furthermore, when blindfolded heterosexual men were caressed by a woman, they responded with pleasure, while if they were told they were being caressed by a man (even if the same woman was stroking them), they responded with aversion (Gazzola et al., 2012; Scheele et al., 2014).

Culture also has an important influence regarding attitudes toward touch. For example, in Italy, it is common practice to greet someone with a kiss on both their cheeks or with a hug. On the contrary, in Japan, bowing is the traditional, customary greeting; there is no physical contact between individuals (McDaniel and Andersen, 1998; Finnegan, 2005). Dibiase and Gunnoe (2004) examined both hand and non-hand touches in Italy, the Czechia, and the United States. Results show that Czech men touch more than any other group, while Czech and Italian women and Italian men use non-hand touch more than the other groups. Authors suggest that these results are partially explained by dominance theory, claiming that touching behavior is an expression of dominance. Dominance is associated not only with social status but also with gender, especially in countries where men are still in clearly dominant positions (Dibiase and Gunnoe, 2004).

When considering personal factors, chronic pain, depression, and anhedonia can reduce the hedonic experiences connected to affiliative touch (Pizzagalli et al., 2008; Elvemo et al., 2015; Thomsen, 2015). Even anxiety can reduce pleasantness connected to touch. Wilhelm et al. (2001) demonstrated that high anxiety women, as compared to low anxiety women, following a 2-min touch on the wrist by a male experimenter, reported greater anxiety and embarrassment.

Age is another factor influencing the pleasantness of touch: affective touch is perceived as more pleasant at a young age (Sehlstedt et al., 2016; Croy et al., 2019). Even gender influences attitudes toward touch: in general, women respond more positively than men to touch (Ozolins and Sandberg, 2009). Furthermore, women experience more pleasantness when touched by strangers in a non-sexual way (Hall et al., 2005), while men prefer being touched by women rather than by men (Gazzola et al., 2012; Scheele et al., 2014). Additionally, men avoid touch significantly more than women toward partner, family, and same-sex individuals (Ozolins and Sandberg, 2009).

Lastly, people vary in their base predisposition toward being touched. The touch avoidance construct is an index of a person’s attitude toward touching and being touched (Andersen and Leibowitz, 1978). Touch avoidance reduces the perceived pleasantness of all kinds of touch (Hielscher and Mahar, 2017). This predisposition develops throughout a person’s lifetime and is a stable personality trait (Johansson, 2013). Cultural and biological factors interacting with early experiences with touch within the family influence attitudes toward touch. According to attachment theory, early patterns of tactile behavior, for example, the nature and degree of touch between parents and their child, predict the child’s later tendencies to seek or avoid touching people outside the family (Deethardt and Hines, 1984). Touch defined as positive for children’s physical and psychological development has been described as patting, stroking, holding hands, tickling, hugging, kissing, stroking, and physically guiding the child (Stansbury et al., 2012). Positive touch during childhood is associated with lower levels of depression and higher relationship satisfaction during adolescence and early adulthood (Takeuchi et al., 2010).

Measuring and assessing touch avoidance is important not only for a deeper understanding of human touch but also for the implications touch has on healthcare practices. Touch in nursing provides a deeper connection with patients and can improve the nurse–patient relationship (Bensing et al., 2013; Deledda et al., 2013; Stein-Parbury, 2013). Moreover, there is evidence that social touch improves well-being and physical recovery (Nabi et al., 2013). There is, however, an association between length of service and comfort in using touch aimed at emotional containment; moreover, female and male nurses differ in performing this type of touch (Trifiletti et al., 2017). Even if most of the time social touch is appreciated by patients, an ignored area of investigation is why touch and massage sometimes fail in their soothing function. For instance, hand massage or foot massage in elderly care sometimes reduce agitation and improve well-being, but sometimes elicit opposite responses. These different responses seem to be associated with lack of experience or lack of confidence with the person who administered the massage, and with gender-mediated responses (Snyder et al., 1995; Moyle et al., 2014). Measuring attitudes toward touch could be important for helping young healthcare professionals to explore their automatic responses in using relational and caring touch, for better understanding when social and comforting touch is efficient and beneficial for patients, and when, in touch avoidant individuals, touch causes anxiety and aversion.

Assessing and measuring touch avoidance could also be helpful in psychotherapeutic settings (Perrella, 2017; Rohner et al., 2019). Clinicians often meet people struggling with romantic relationships, sexuality, or assertiveness. Often, clinicians’ focus is on top–down processes: beliefs, motivations, and expectations that influence the patient’s approach to other people and the patient’s own responses. However, this approach may ignore how basal attitudes toward touch influence their patient’s interpersonal relationships, social contexts, and bonding. Additionally, on the part of the patient, it can be useful to increase awareness and acceptance of their automatic responses to touch, experiencing their sensations and bodies in a non-judgmental way. This may help them inhibit automatic and dysfunctional responses to their unpleasant physical reactions, which could lead them to avoid meaningful relationships altogether. The role of touch in shaping and maintaining relationships has been explored in many studies of attachment. Touch seems strictly related to attachment style, a useful measure to predict how people experience intimate relationships during adulthood. Greenspan and Bowlby (1974) claimed that human touch facilitates the bond between a child and their caregiver and is essential for the child’s well-being, especially in their early years. Anxious or avoidant individuals report a higher level of touch avoidance (Anderson, 1987; Brennan et al., 1998). Moreover, anxious individuals avoid intimacy, while dismissing individuals avoid closeness (Bartholomew and Shaver, 1998). Nelson and Geher (2007) showed that individuals who scored higher on anxiety subscales of an attachment questionnaire (worried style) reported more frequent cuddle-seeking behavior. Finally, individuals avoiding touch seem to need more intimacy in relationship, as a proof of their partner’s love (Johansson, 2013).

To measure touch avoidance, Andersen and Leibowitz (1978) built a self-report instrument: the Touch Avoidance Measure (TAM), assessing attitudes toward and comfort with touch. The TAM is one of the most frequently used scales for measuring this trait. The scale analyzes two dimensions of touch avoidance: same-sex touch avoidance and opposite-sex touch avoidance. Another, more recent instrument is the Touch Avoidance Questionnaire (TAQ), developed by Ozolins and Sandberg (2009) a wide-ranging instrument that measures attitudes toward touch. The scale includes items that assess several social contexts of touch, including touch with a romantic partner, family (parents, siblings), same- and opposite-sex friends, and with strangers.

The aim of this study is to translate and validate in Italian these two questionnaires.

## Materials and Methods

### Procedure

The study involved 335 participants (216 female, 113 male, six undisclosed; age = 35.82 ± 14.32; range, 16–74) recruited through convenience sampling drawing on the authors’ personal networks. Participation was voluntary and anonymous, and participants received no compensation. The presentation order of the tests (all pencil-and-paper) was counterbalanced. Forty-one participants (30 female, eight male, three undisclosed; age, 27.84 ± 9.30; range, 20–55) were contacted a week after to do the test–retest, and 1 month after the first administration of the test to complete again the TAM and the TAQ.

Estimating an adequate sample size for a CFA depends on several aspects, including factor loadings, number of indicators per factor, estimator used, and the fit indices being considered (Kyriazos, 2018). Common, conservative rules of thumb for choosing adequate sample size for a CFA include ensuring that *N* > 300 (Tabachnick and Fidell, 2012) and that the ratio *N*/*p* (where *p* is the number of indicator variables) is above 10 (Wang and Wang, 2012). Additionally, Tinsley and Tinsley (1987) suggested that an *N*/*p* ratio between 5 and 10 can be adequate when *N* > 300. In our case, the *N*/*p* ratio is 18.6 for the TAM and 10.8 for the TAQ. All missing data (1.9% for the TAM and 2.4% for the TAQ) were handled through pairwise deletion when estimating the correlation matrices.

Sample size was also adequate for the test–retest reliability estimates, following Hertzog (2008) recommendations.

The Ethics Committee of the University of Campania “Luigi Vanvitelli,” Department of Psychology, approved this study. Recruiting and testing conformed with the local Ethics Committee requirements and the Declaration of Helsinki.

### Measures

In 1978, Andersen and Leibowitz created the TAM to assess attitudes toward touch and comfort levels regarding touch. This instrument is composed of 18 statements concerning feelings about touching other people and being touched. Participants answer each item by rating it on a one- to five-point Likert scale (1 “fully disagree”; 5 “fully agree”). The measure is composed of two subscales: same sex (10 items, e.g., “Touching a friend of the same sex does not make me uncomfortable”) and opposite sex (eight items, e.g., “When a member of the opposite sex touches me, I find it unpleasant”).

In 2009, Ozolins and Sandberg developed the TAQ in order to assess level of touch avoidance in different contexts, such as situations involving romantic partner, siblings, parents, friends, professional touch, and touch with complete strangers. Some of the questions concerned attitudes toward touch with a same-sex friend and some with a friend of the opposite sex. Across these categories, there were different questions about touching and receiving touch. The TAQ consists of 31 Likert-type items to which participants are asked to respond on a five-point scale (1 “fully disagree”; 5 “fully agree”). Items are divided in the subscales Partner (10 items, e.g., “I wish my partner would hold me for hours”), Family (six items, e.g., “I grew up in a cuddly family”), Same sex (six items, e.g., “I like to hug a same-sex friend”), opposite-sex (six items, e.g., “I try to avoid touch with an opposite-sex friend”), and Stranger [three items, e.g., “I find it very unpleasant to be in contact with unknown people (e.g., in queues, on the bus)”].

We translated the two scales from English to Italian; subsequently, an English native language speaker proceeded with the back translation; finally, we asked the authors of the TAM and the TAQ if the back translation was adequate. The Italian version of the questionnaires is included in Supplementary Material.

### Data Analysis

Overall, questionnaire responses indicated a high degree of non-normality for both the TAM and the TAQ (see Table 1) and the presence of considerable ceiling/floor effects. Mardia’s tests for multivariate skew and kurtosis are significant for both the TAM (skew = 2738.44, *p* < 0.001, kurtosis = 21.04, *p* < 0.001) and the TAQ (skew = 14826.86, *p* < 0.001, kurtosis = 30.56, *p* < 0.001). For this reason, we opted to treat the items as ordinal, fitting confirmatory factorial analyses (CFAs) that would not be biased by the skewed distribution of data. However, a simple CFA with weighted least square mean and variance adjusted estimator would not converge due to: (1) the high number of parameters, since the model would require estimation of four threshold parameters for each item, and (2) the lack of observations for some response categories (i.e., 5 or 1) for some of the items due to ceiling/floor effects. Therefore, before fitting the CFAs, we recoded item responses as “low” (1 or 2), “medium,” (3) or “high” (4 or 5). This would require the estimation of only two thresholds for each item, increasing the degrees of freedom and thus reducing the risk of fit indexes inflation due to overparameterization. Moreover, after recoding, all response categories had data, allowing CFA models to converge. For evaluating goodness of fit, we followed Hu and Bentler’s (1999) conservative recommendations of comparative fit index (CFI) and Tucker–Lewis index (TLI) > 0.95 and root-mean-square error of approximation (RMSEA) < 0.06 as indicating excellent fit, guidelines widely followed in modern SEM research (Boduszek et al., 2016; Willmott et al., 2018).

**TABLE 1:** Descriptive statistics for Touch Avoidance Measure (TAM) and Touch Avoidance Questionnaire (TAQ) items.

	Mean	SD	Median	Skew	Kurtosis		Mean	SD	Median	Skew	Kurtosis
TAQ_1	3.51	1.09	4	–0.52	–0.58	TAQ_31	3.86	0.85	4	–1.26	2.39
TAQ_2	1.87	0.86	2	1.19	1.73	TAQ_32	3.74	0.96	4	–0.95	0.72
TAQ_3	1.45	0.89	1	2.35	5.28	TAQ_33	1.94	1.00	2	1.08	0.71
TAQ_4	2.09	1.09	2	0.82	–0.26	TAQ_34	2.00	1.11	2	1.00	0.15
TAQ_5	1.63	0.89	1	1.61	2.40	TAQ_35	2.49	1.09	2	0.38	–0.54
TAQ_7	3.47	1.09	4	–0.60	–0.41	TAQ_37	3.39	1.13	4	–0.32	–0.70
TAQ_8	3.99	0.81	4	–0.66	0.36	TAM_1	4.05	0.88	4	–1.05	1.27
TAQ_9	1.39	0.63	1	1.81	4.21	TAM_2	3.45	0.78	3	–0.49	0.88
TAQ_11	1.86	1.02	2	0.99	–0.15	TAM_3	3.15	1.12	3	–0.11	–1.02
TAQ_14	2.03	1.05	2	0.82	–0.06	TAM_4	1.43	0.76	1	2.04	4.28
TAQ_16	3.38	1.28	4	–0.36	–1.00	TAM_5	3.28	1.02	3	–0.61	–0.21
TAQ_17	3.10	1.29	3	–0.04	–1.14	TAM_6	3.99	0.84	4	–0.90	1.14
TAQ_18	3.65	1.16	4	–0.73	–0.27	TAM_7	1.67	0.86	1	1.37	1.71
TAQ_19	2.78	1.26	3	0.14	–1.08	TAM_8	1.91	0.99	2	1.08	0.66
TAQ_20	2.74	1.35	3	0.14	–1.34	TAM_9	3.31	1.12	3	–0.41	–0.53
TAQ_21	2.20	1.28	2	0.81	–0.55	TAM_10	2.94	1.17	3	–0.14	–0.91
TAQ_22	1.82	1.00	2	1.34	1.27	TAM_11	2.19	1.29	2	0.74	–0.67
TAQ_23	3.62	1.07	4	–0.83	0.16	TAM_12	2.98	1.13	3	–0.31	–0.79
TAQ_24	1.93	1.03	2	1.10	0.60	TAM_13	3.78	1.01	4	–1.08	0.97
TAQ_25	3.94	0.86	4	–1.22	2.46	TAM_14	4.74	0.51	5	–2.04	5.00
TAQ_26	3.71	0.98	4	–0.89	0.63	TAM_15	3.78	1.09	4	–0.73	–0.16
TAQ_27	1.94	1.01	2	1.16	0.97	TAM_16	1.96	1.00	2	1.19	1.18
TAQ_28	2.22	1.10	2	0.68	–0.48	TAM_17	3.93	1.12	4	–1.14	0.59
TAQ_29	3.65	0.97	4	–0.78	0.26	TAM_18	2.27	1.09	2	0.72	–0.21
TAQ_30	2.03	1.05	2	0.91	0.04						

Touch Avoidance Questionnaire items 6, 10, 12, 13, 15, and 36 were excluded from the final version of the test by the original authors (Ozolins and Sandberg, 2009).

After confirming the original models, the reliability of the scales was measured using ordinal alpha for each subscale of the tests (Gadermann et al., 2012) as well as test–retest reliability after 1 week and 1 month.

Subsequently, we tested convergent validity by computing the correlations between the Same Sex subscales of the two tests, and between their Opposite Sex subscales.

All data analyses were conducted using R (Version 3.6.2).

## Results

### Confirmatory Factor Analysis

For both questionnaires, we tested the models conceptualized for their respective original versions, i.e., a two-factor model for the TAM (attitudes toward same sex touch and opposite sex touch) and a five-factor model for the TAQ (attitudes toward touch with one’s partner, their same/opposite sex friends, their family, and strangers).

We observed good, but not excellent, fit for the TAM [CFI = 0.947, TLI = 0.940, RMSEA = 0.065, standardized root-mean-square residual (SRMR) = 0.138]. All factor loadings were statistically significant, and their sign was the same as in the original scale. Correlation between the two factors was 0.56. Examination of model parameters identified a relatively low loading for item 14 (0.176, see Table 2). The loading is significantly above 0, but the estimate is below the often-used cutoff of 0.300. Additionally, examination of the highest modification indexes suggested adding correlations between the errors of items 7 and 8 and adding a loading for item 2 on the Same Sex factor. We did not apply these model modifications. Goodness-of-fit measures, even if slightly below Hu and Bentler’s suggested cutoffs, still indicate relatively good fit. When considering the trade-off implicated in improving goodness of fit by editing the model, making comparisons with studies employing the English version of the TAM impossible, we deemed it better to err on the side of preserving the original model. This decision was also informed by the lack of theoretical grounding for adding the loading of item 2 on the Same Sex factor.

**TABLE 2:** Factor loadings for the Touch Avoidance Measure (TAM) and Touch Avoidance Questionnaire (TAQ).

Item	Factor	Loading	*p*	Item	Factor	Loading	*p*
TAQ_1	TAQ Partner	0.798	<0.001	TAQ_31	TAQ Opposite Sex	0.691	<0.001
TAQ_2	TAQ Partner	–0.649	<0.001	TAQ_32	TAQ Opposite Sex	0.904	<0.001
TAQ_3	TAQ Partner	–0.481	<0.001	TAQ_33	TAQ Opposite Sex	–0.854	<0.001
TAQ_4	TAQ Partner	–0.610	<0.001	TAQ_34	TAQ Stranger	0.541	<0.001
TAQ_5	TAQ Partner	–0.796	<0.001	TAQ_35	TAQ Stranger	0.826	<0.001
TAQ_7	TAQ Partner	0.851	<0.001	TAQ_37	TAQ Stranger	0.448	<0.001
TAQ_8	TAQ Partner	0.548	<0.001	TAM_1	TAM Same Sex	0.409	<0.001
TAQ_9	TAQ Partner	–0.787	<0.001	TAM_2	TAM Opposite Sex	0.740	<0.001
TAQ_11	TAQ Partner	–0.601	<0.001	TAM_3	TAM Same Sex	0.509	<0.001
TAQ_14	TAQ Partner	–0.330	<0.001	TAM_4	TAM Same Sex	–0.660	<0.001
TAQ_16	TAQ Family	0.824	<0.001	TAM_5	TAM Opposite Sex	0.841	<0.001
TAQ_17	TAQ Family	0.842	<0.001	TAM_6	TAM Same Sex	0.655	<0.001
TAQ_18	TAQ Family	0.712	<0.001	TAM_7	TAM Opposite Sex	–0.738	<0.001
TAQ_19	TAQ Family	0.732	<0.001	TAM_8	TAM Opposite Sex	–0.505	<0.001
TAQ_20	TAQ Family	–0.692	<0.001	TAM_9	TAM Same Sex	0.743	<0.001
TAQ_21	TAQ Family	–0.817	<0.001	TAM_10	TAM Opposite Sex	0.672	<0.001
TAQ_22	TAQ Same Sex	–0.748	<0.001	TAM_11	TAM Same Sex	0.607	<0.001
TAQ_23	TAQ Same Sex	0.773	<0.001	TAM_12	TAM Same Sex	0.887	<0.001
TAQ_24	TAQ Same Sex	–0.799	<0.001	TAM_13	TAM Same Sex	0.666	<0.001
TAQ_25	TAQ Same Sex	0.603	<0.001	TAM_14	TAM Opposite Sex	0.176	0.024
TAQ_26	TAQ Same Sex	0.871	<0.001	TAM_15	TAM Opposite Sex	0.716	<0.001
TAQ_27	TAQ Same Sex	–0.830	<0.001	TAM_16	TAM Same Sex	–0.479	<0.001
TAQ_28	TAQ Opposite Sex	–0.731	<0.001	TAM_17	TAM Opposite Sex	0.542	<0.001
TAQ_29	TAQ Opposite Sex	0.827	<0.001	TAM_18	TAM Same Sex	–0.562	<0.001
TAQ_30	TAQ Opposite Sex	–0.923	<0.001				

The confirmatory factor analysis for the TAQ, instead, achieved excellent fit, except for SRMR (CFI = 0.954, TLI = 0.950, RMSEA = 0.058, SRMR = 0.125). All factor loadings were statistically significant and above—in absolute value—the cutoff of 0.300 (see Table 2). The signs of all loadings were the same as in the original scale. Correlations between TAQ factors are reported in Table 3.

**TABLE 3:** Correlations between Touch Avoidance Questionnaire (TAQ) factors.

	Partner	Same Sex	Opposite Sex	Family	Stranger
Partner	1	0.29	0.13	0.26	–0.30
Same Sex	0.29	1	0.53	0.29	–0.45
Opposite Sex	0.13	0.53	1	0.13	–0.61
Family	0.26	0.29	0.13	1	–0.40
Stranger	–0.30	–0.45	–0.61	–0.40	1

Models for the two questionnaires were fitted separately. TAQ items 6, 10, 12, 13, 15, and 36 were excluded from the final version of the test by the original authors (Ozolins and Sandberg, 2009).

### Reliability

Ordinal alpha was satisfactory for all but one of the subscales of the TAM and the TAQ. Specifically. for the TAM, we observe α = 0.85 (0.82, 0.87) for the Same Sex subscale and α = 0.84 (0.81, 0.87) for the Opposite Sex subscale (in the original validation paper, α = 0.82 and 0.88, respectively). Ordinal α for the whole scale is 0.88 (0.86, 0.90). The high internal consistency for the whole scale is not only due to inflation of α for scales with more items (Kopalle and Lehmann, 1997) but also due to high correlation between the two TAM factors (0.559).

For the TAQ, we observe an ordinal α = 0.84 (0.81, 0.87) for the Partner subscale, ordinal α = 0.89 (0.86, 0.91) for the Same Sex subscale, ordinal α = 0.92 (0.90, 0.93) for the Opposite Sex subscale, ordinal α = 0.88 (0.86, 0.91) for the Family subscale, and ordinal α = 0.59 (0.54, 0.70) for the Stranger subscale. The low value of α for the latter subscale may be because the subscale consists of only three items and α is sensitive to scale length. However, Spearman–Brown “prophecy” formula (Brown, 1910; Spearman, 1910) would predict an α as low as 0.74 were to subscale to comprise six items, suggesting that the Stranger subscale does have relatively low internal consistency. Ordinal α for the whole TAQ is 0.89 (0.85, 0.91). For comparison, in the original validation paper, αs were 0.86 (Partner), 0.89 (Same Sex), 0.85 (Opposite Sex), 0.85 (Family), and 0.64 (Stranger).

Test–retest reliability was examined by computing Pearson’s correlation for the factor scores measured at each measurement time. Reliability was satisfactory for all subscales, ranging from 0.80 to 0.94 for 1-week retest, and from 0.67 to 0.90 for 1-month retest (see Table 4). All correlations are significant for *p* < 0.001.

**TABLE 4:** Test–retest correlation after 1 week and 1 month for all Touch Avoidance Measure (TAM) and Touch Avoidance Questionnaire (TAQ) subscales.

Subscale	1 Week test–retest Pearson’s correlation	1 Month test–retest Pearson’s correlation
TAM Same Sex	0.91 (0.84, 0.96)	0.87 (0.75, 0.93)
TAM Opposite Sex	0.80 (0.63, 0.89)	0.70 (0.47, 0.84)
TAQ Partner	0.82 (0.70, 0.91)	0.81 (0.61, 0.91)
TAQ Same Sex	0.88 (0.77, 0.94)	0.87 (0.73, 0.94)
TAQ Opposite Sex	0.81 (0.64, 0.90)	0.67 (0.39, 0.84)
TAQ Family	0.94 (0.88, 0.97)	0.90 (0.79, 0.95)
TAQ Stranger	0.89 (0.78, 0.94)	0.82 (0.65, 0.92)

### Convergent Validity

The correlation between the Same Sex subscales of the TAM and TAQ is 0.72 (0.66, 0.77), *p* < 0.001. The correlation between the Opposite Sex subscales for the two questionnaires is 0.63 (0.56, 0.70), *p* < 0.001. These results are in line with the TAQ validation study (Ozolins and Sandberg, 2009) which found correlations of 0.62 and 0.57 for the Same Sex and Opposite Sex scales, respectively. Inspection of item content reveals different conceptualization of the factors between the two tests: the TAQ always investigates attitudes toward being touched by friends of the same (or opposite) sex; the TAM, instead, usually refers to “people” of the same (opposite) sex, therefore investigating attitudes toward being touched by strangers.

**FIGURE 1:**
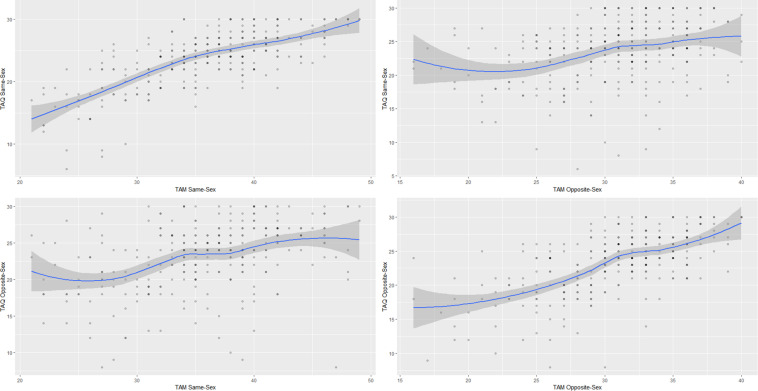
Scatterplots for the Same Sex and Opposite Sex subscales of the Touch Avoidance Measure (TAM) and Touch Avoidance Questionnaire (TAQ). The polynomial regression curve was added to show the approximate linearity of relationship between subscales. The shaded area represents the 95% confidence area for the regression curve.

The correlation between the Same Sex subscale of the TAM and the Opposite Sex subscale of the TAQ is 0.40 (0.30, 0.49), *p* < 0.001, while the correlation between the Opposite Sex subscale of the TAM and the Same Sex subscale of the TAQ is 0.34 (0.23, 0.43), *p* < 0.001. These magnitudes seem to be consistent with those that would be expected for correlated constructs measured with different questionnaires adopting slightly different definitions. See Figure 1 for the scatterplots between these subscales.

## Discussion

In this study, we translated and validated two measures of touch avoidance—the TAM and the TAQ—for use on the Italian population. Overall, both validations can be considered successful: both the TAM and the TAQ models achieved good fit, 1-week and 1-month test–retest reliabilities are high, and convergent validity results are in the expected direction and magnitude. However, there are a few caveats that should be considered when using the TAM and TAQ with Italian samples.

First and foremost, goodness-of-fit indexes for the TAM were below Hu and Bentler (1999) cutoffs for excellent fit, and inspection of model parameters and modification indexes suggested to slightly modify the original model. We opted not to enact these modifications, as the model fit was still relatively good, with the rationale of providing Italian researchers with an instrument as close as possible to the original one for comparison and legacy purposes. However, we strongly suggest to use the TAQ, unless there are compelling reasons to use the TAM (e.g., for the replication of a study that used the TAM). This suggestion is borne not only of statistical considerations: TAM’s domain is narrower than TAQ’s, as it investigates touch avoidance only in the case of same and opposite sex people, without considering the additional settings investigated by the TAQ (strangers, family, partner). Additionally, some of TAM’s items do appear a little dated, either in formulation or due to the changes in societal norms that occurred between 1978 and today.

Employing the TAQ therefore seems to be preferred, although it should be kept in mind that its “Stranger” subscale appears to have relatively low internal consistency and Opposite Sex subscale may have lower test–retest reliability than the others. Despite these minor concerns, the scale can be useful for investigating attitudes toward touch either in healthcare or in psychotherapeutic settings, so as to inform practitioners in the best course of action with their patients.

Subsequent studies could focus on investigating how touch avoidance varies in the Italian population according to gender, region of origin, age, or personality traits. Additionally, testing for measurement invariance for age and gender, as well as between the English and Italian versions of the tests could improve comparability of results across different populations.

## Data Availability Statement

The datasets presented in this study can be found in online repositories. The names of the repository/repositories and accession number(s) can be found below: https://github.com/M-Pass/Touch-Avoidance-data.

## Ethics Statement

The studies involving human participants were reviewed and approved by the Ethics Committee of the University of Campania “Luigi Vanvitelli,” Department of Psychology. The patients/participants provided their written informed consent to participate in this study.

## Author Contributions

All authors conceived and designed the study, organized and supervised data collection and imputing, drafted the manuscript, organized and supervised the data analysis, and read and agreed to the published version of the manuscript.

## Conflict of Interest

The authors declare that the research was conducted in the absence of any commercial or financial relationships that could be construed as a potential conflict of interest.
