# Supplementary material for: Italian Validation of the Touch Avoidance Measure and the Touch Avoidance Questionnaire
Source: Front Psychol. 2020 Jul 23;11:1673. doi: 10.3389/fpsyg.2020.01673 (PMC7390897; doi:10.3389/fpsyg.2020.01673)
Supplement: Supplementary file 1 [file Table_1.DOCX]

# **Supplementary Materials for the Italian validation of the Touch Avoidance Measure and the Touch Avoidance Questionnaire**

Table SM1.

Items of the Italian version of the Touch Avoidance Measure. Items marked * must be reversed before scoring.

| Item | Subcale | Italian translation |
| --- | --- | --- |
| 1* | Same-sex | Un abbraccio da parte di un amico del mio stesso sesso è un vero segno di amicizia. |
| 2* | Opposite-sex | Gli amici/amiche del sesso opposto al mio apprezzano quando li tocco. |
| 3* | Same-sex | Spesso metto un braccio attorno alle spalle degli amici dello stesso sesso. |
| 4 | Same-sex | Mi rivolta vedere persone dello stesso sesso che si abbracciano. |
| 5* | Opposite-sex | Mi piace che persone del sesso opposto al mio mi tocchino. |
| 6* | Same-sex | Le persone non dovrebbero sentirsi così tese nel toccare individui dello stesso sesso. |
| 7 | Opposite-sex | Penso che sia volgare che persone del sesso opposto al mio mi tocchino. |
| 8 | Opposite-sex | Trovo spiacevole quando una persona del sesso opposto mi tocca. |
| 9* | Same-sex | Mi piacerebbe essere libero di dimostrare le mie emozioni alle persone dello stesso sesso toccandole. |
| 10* | Opposite-sex | Mi piacerebbe massaggiare un amico del sesso opposto. |
| 11* | Same-sex | Mi piace baciare persone dello stesso sesso. |
| 12* | Same-sex | Mi piace toccare gli amici del mio stesso sesso. |
| 13* | Same-sex | Toccare un amico dello stesso sesso non mi mette a disagio. |
| 14* | Opposite-sex | Trovo piacevole abbracciare il mio partner. |
| 15* | Opposite-sex | Mi piace ricevere massaggi sulla schiena da qualcuno del sesso opposto al mio. |
| 16 | Same-sex | Non mi piace baciare i parenti del mio stesso sesso. |
| 17* | Opposite-sex | Il contatto fisico intimo con persone del sesso opposto al mio è piacevole. |
| 18 | Same-sex | Trovo difficile essere toccato da un membro del mio stesso sesso. |

Table SM2.

Items of the Italian version of the Touch Avoidance Questionnaire. Items marked * must be reversed before scoring. Items marked ** are not included in the scoring.

| Item | Subscale | Italian translation |
| --- | --- | --- |
| 1* | Partner | Vorrei che il mio partner mi stringesse per ore |
| 2 | Partner | Spesso devo dire al mio partner di smettere di toccarmi |
| 3 | Partner | Quando il mio partner mi tocca provo una sensazione spiacevole per la maggior parte del tempo |
| 4 | Partner | Il mio partner spesso si lamenta che non lo/la tocco abbastanza |
| 5 | Partner | Talvolta cerco qualche scusa per evitare di toccare il mio partner |
| 6** | - | Mi piacerebbe che il mio partner mi toccasse più spesso |
| 7* | Partner | Mi piace abbracciare e accarezzare il mio partner per ore |
| 8* | Partner | Sono sempre felice quando il mio partner mi tocca |
| 9 | Partner | Spesso trovo che sia insopportabile l'essere toccato/a dal mio partner |
| 10** | - | Mi rattrista che il mio partner non mi voglia toccare come vorrei |
| 11 | Partner | Qualche volta ritengo che essere toccato dal mio partner sia irritante |
| 12** | - | Ci metto un po' ad abituarmi a essere toccato dal mio partner se siamo stati lontani per un periodo di tempo |
| 13** | - | Ho pensato di interrompere la relazione col mio partner perché non gli/le piaceva essere toccato/a |
| 14 | Partner | Non voglio che il mio partner mi tocchi in pubblico |
| 15** | - | Qualsiasi tipo di contatto fisico con i parenti del mio partner non mi piace per nulla |
| 16* | Family | Abbraccio/ho abbracciato sempre mia madre quando la vedo/vedevo |
| 17* | Family | Abbraccio/ho abbracciato sempre mio padre quando lo vedo/vedevo |
| 18* | Family | Sono cresciuto coccolato/a nella mia famiglia |
| 19* | Family | C'è molto contatto fisico tra me e i miei fratelli |
| 20 | Family | Sono stato raramente abbracciato da mio padre |
| 21 | Family | Sono stato raramente abbracciato da mia madre |
| 22 | Same-sex | Di solito trovo difficile abbracciare un amico dello stesso sesso |
| 23* | Same-sex | Mi piace abbracciare un amico del mio stesso sesso |
| 24 | Same-sex | Di solito cerco di evitare di toccare o di essere toccato da un amico del mio sesso |
| 25* | Same-sex | Mi piace sedermi vicino a un amico del mio stesso sesso |
| 26* | Same-sex | Mi piace quando un amico del mio stesso sesso mi abbraccia |
| 27 | Same-sex | Non mi piace per niente quando un amico del mio stesso sesso mi tocca |
| 28 | Opposite-sex | Spesso trovo difficile toccare un amico del sesso opposto al mio |
| 29* | Opposite-sex | Mi piace abbracciare un amico del sesso opposto al mio |
| 30 | Opposite-sex | Di solito cerco di evitare di toccare o di essere toccato da un amico del sesso opposto al mio |
| 31* | Opposite-sex | Mi piace sedermi vicino a un amico del sesso opposto al mio |
| 32* | Opposite-sex | Mi piace quando un amico del sesso opposto al mio mi abbraccia |
| 33 | Opposite-sex | Non mi piace per niente quando un amico del sesso opposto al mio mi tocca |
| 34 | Stranger | Non mi piace il contatto fisico con i bambini delle altre persone |
| 35 | Stranger | Spesso trovo che toccare o essere toccati sia un gesto invadente |
| 36** | - | Trovo che il contatto fisico da parte di professionisti (parrucchiere, massaggiatore, ...) sia piacevole |
| 37 | Stranger | Trovo molto spiacevole avere un contatto fisico con persone sconosciute (ad es. in coda, nell'autobus...) |
